# Post-flood communicable disease surveillance following the 2023 dam collapse in Eastern Libya

**DOI:** 10.1371/journal.pgph.0007124

**Published:** 2026-08-20

**Authors:** Salem Alkoshi, Attayeb Mohsen

**Affiliations:** 1 Faculty of Health Sciences, Al Asmarya Islamic University, Zliten, Libya; 2 Libyan Centre for Dental Research, Zliten, Libya; PLOS: Public Library of Science, UNITED STATES OF AMERICA

## Abstract

Tropical Storm Daniel struck northeastern Libya on 10 September 2023, leading to the collapse of two dams and catastrophic floods. The disaster resulted in massive casualties and infrastructure damage, and led to infectious disease outbreaks. This study aimed to summarize the incidence and distribution of infectious diseases reported after the flood, describe temporal and geographical trends, and public health implications. We retrospectively reviewed 90 daily epidemiological reports produced by the National Routine Surveillance System (NRSS) between 14 September and 24 December 2023. From each report, we extracted the number of new cases of infectious diseases recorded by district and age group. Population-standardized incidence rates were computed per 100,000 population at the district level to observe temporal trends across the ecological unit for all infectious diseases according to Libyan infectious disease surveillance guideline, moreover weekly moving averages, and geographic distribution maps were drawn. A total of 27,368 infectious disease cases representing 15 different diseases were reported. Respiratory infections (14,031 cases; 701.67/100,000 population) and diarrhea (12,082 cases; 604.21/100,000 population) were the most common. The surveillance also identified skin-related conditions (chickenpox and rashes), vaccine-preventable diseases (measles and rubella), and vector-borne illnesses (leishmaniasis and malaria). Diarrheal cases peaked around 42 days post-flood, then declined following a local educational intervention promoting safe water practices. Geographically, Benghazi had the highest respiratory infection count, while Albayda reported the most diarrheal cases. Notably, high-threat pathogens such as cholera were not detected, and the number of malaria and measles cases remained minimal. The peak of diarrheal diseases occurred six weeks post-flood, then declined following educational intervention. Results suggest that disaster preparedness, safe water supply, and enhanced surveillance capacity are essential. These results highlight the consequences of limited resources and weak infrastructure preparedness on the spread of infectious diseases.

## Introduction

Floods are devastating natural disasters, having impacted more than two billion people worldwide between 1998 and 2017 [1] and causing widespread damage to infrastructure, health systems, and livelihoods [2]. In the period from 2020 to 2025, floods caused 37,214 deaths, impacted 232 million people, and cost an estimated 268 billion USD globally [3].

During floods, the risk of infection increases (waterborne, vector-borne, and respiratory infections) because of disrupted sanitation, contaminated water sources, and population displacement [4,5]. Diarrheal diseases affect flood victims, especially those in poor living conditions, and pose a major challenge to public health systems [6–9]. Outbreaks of diarrhea often occur within weeks to months after flooding, particularly in impoverished neighborhoods with inadequate water supply. Multiple pathogens are implicated, including cholera, rotavirus, typhoid, and paratyphoid [2,6,7,10,11]. Moreover, flood-related diseases are exacerbated by the substandard living conditions of internally displaced people (IDP) in crowded shelters [2,6,10,12].

Flood exposure also leads to other health problems such as bacterial and viral respiratory infections, hepatitis A and E, cutaneous infections, leptospirosis, and mosquito-borne illnesses. Beyond that, psychological disorders are also a significant consequence of flood, though they are non-communicable [6,13].

In Asia, flooding of the Huai River in China generally causes a massive disaster, and spreads several infections such as malaria, diarrhea, and bacillary dysentery [14]. Pakistan suffered from massive floods in 2010 and 2022, which affected millions of people and caused severe respiratory infections, bloody diarrhea, cholera, cryptosporidiosis, rotavirus infections, typhoid and paratyphoid fevers, and outbreaks of poliomyelitis [14,15]. Furthermore, cholera is prevalent in Bangladesh post-flood [16], and in India during the monsoon season [14].

Displacement and loss of property during floods is a risk factor for several infections, including measles and tetanus outbreaks in areas with low vaccination coverage, as well as meningitis, acute respiratory infections, leptospirosis, malaria, and dengue. Hepatitis A and E are common after frequent floods, especially with limited access to safe water and sanitation [16,17].

In Latin America, Brazil, Colombia, Peru, and Ecuador have suffered the consequences of catastrophic flooding, leading to outbreaks of infectious diseases such as leptospirosis and dengue, as well as waterborne and vector-borne diseases. Open sewers posed a 42% risk of leptospirosis infection among residents living nearby during floods [18,19].

In sub-Saharan Africa, flooding is a known risk factor for water-borne, vector-borne, and zoonotic diseases such as malaria, cholera, scabies, and tapeworm disease [20]. Exposure to flooding has been strongly linked to increased hospital admissions for post-flood illnesses [21].

Like many Mediterranean and North African countries, Libya is increasingly vulnerable to extreme climate events due to aging infrastructure and limited early-warning capabilities. Ninety-five percent of Libya’s total area has a desert climate, which is exacerbated by very few rainy days per year. Consequently, this has forced people to rely on groundwater for drinking. Moreover, the desertification and extensive utilization of these non-renewable water sources lead to increased stress on them that may cause salinization and progressively reduced water quality [22].

Residing in flood-prone areas—especially where early warning systems and flood-safety education are lacking—greatly increases the risk of flood-related harm [1,6]. Moreover, Libya suffered from the consequences of prolonged internal and external conflicts following the Arab Spring uprising in 2011, which destroyed many healthcare facilities and water-related infrastructure. The life expectancy of the Libyan population has dramatically decreased after the conflict. These conflicts continued, leading to further infrastructure damage and the collapse of access to the health care system [23]. The affected water-related infrastructure, such as dams and desalination stations, further exacerbates the country’s humanitarian and environmental burden [24,25].

In Libya, on September 10, 2023, Storm Daniel dropped an average of 350 mm of rainfall within 24 hours, exceeding the capacity of local dams. The subsequent failure of the Bu Mansour and Al-Bilad dams released catastrophic flood waves that engulfed the city, resulting in the death of 20% of Derna’s population [26]. This was exacerbated by the presence of an old dam system that had been poorly maintained and inadequately designed from the outset. A few years before the event, defects were detected in both dams (subsidence and serious tunneling). They were serious findings that required immediate maintenance and repair; however, due to the country’s instability and military conflict, these problems were not solved [24]. Furthermore, the disaster triggered a complex public health crisis characterized by mass displacement and infrastructure collapse across Derna and other cities in the northeast of Libya [27].

While the Libyan National Center for Disease Control (NCDC) prepared numerous reports to track various disease outbreaks, these reports have not yet been systematically analyzed or summarized. This study aimed to synthesize daily surveillance data from the NCDC to identify infectious diseases emerging after the 2023 floods, quantify their incidence and trends to guide future public health preparedness and emergency responses in flood-affected regions.

Despite existing research on the consequences of floods and dam collapses, there is a significant gap in the high-resolution, longitudinal data concerning these events in North Africa. This study is also unique in its extended 90-day examination of disaster effects.

A novel aspect of this study is the utilization the National Routine Surveillance System (NRSS) to track disease prevalence across geographic regions and time, and highlight the key events. These results will inform best preparedness procedures and identify areas of deficiency to be addressed.

## Methodology

### Affected area

Libya is located in the North African region, and it is bordered by the Mediterranean Sea to the north, Egypt and Sudan to the east, Tunisia and Algeria to the west, and Chad and Niger to the south. It has a vast area of 1.7 million square kilometers, and an extended coastline of around 1700 km. Most of the country is characterized by an arid desert climate. The primary source of national income is the petroleum industry. Further information is extensively described in the literature [22,24].

The districts affected by the heavy rain and flood and covered by the active infectious disease surveillance are Albayda, Almarj, Benghazi, Derna, Ejdabia and Tobroq. The total affected area exceeds 261,000 square km [28], with a population of 1,999,646. The districts span from 18.46° W to 25.00° E longitude, and from 32.93° N to 27.01° S latitude. Further information about the geographical context, including the locations and history of the affected dams, and the relevant watershed systems are available in literature [24,29].

### Study design and data source

This retrospective descriptive study was conducted using aggregated data from the National Routine Surveillance System (NRSS) operated by the Libyan NCDC collected between 14 September and 24 December 2023 [30].

This active surveillance system was triggered by the collapse of the dams and monitored the communicable diseases that are mandatorily reported as per the Libyan law No. 106 of 1973 (the Health Code) [31]. The data were provided by certified local surveillance officers and experts responsible for reporting diseases in both emergency and routine public health contexts.

The dataset consisted of 90 daily epidemiological reports generated over the three-month post-flood period. Weekend data were consolidated into the subsequent working day’s report. Each report included demographic and epidemiological detailed variables, including date, city, case counts, age stratification (under or above 5 years old), and disease type. All reports were included in this study’s analysis.

The NRSS reports contained (1) a narrative summary of field surveillance activities and (2) a surveillance table listing communicable disease case counts categorized by city and age group. New daily case counts were extracted from these tables and stratified by city and age group.

The NRSS relies on official public health officers and physicians to submit the data in a paper format from all public-sector hospitals and health centers that provide emergency health services.

Due to the limited public health laboratory resources, a small subset of patient samples was transferred to the infectious diseases laboratory established after the disaster in Derna.

Written authorization was obtained from the Research and Documentation Office (Reference No. 94436/11-6) on 20 October 2024. This study is a secondary analysis of publicly available government reports; hence, formal ethical and Institutional Review Board (IRB) approval were not required.

### Data analysis

The total number of cases was summarized in tables and plotted as histograms. A seven-day moving average was calculated and plotted with means and confidence intervals. All analyses were performed in R (v4.3.1) using the dplyr and ggplot2 packages. Area-specific population estimates obtained from the Bureau of Statistics and Census (BSC) (2023) [32] were used to calculate incidence rates per 100,000 inhabitants. Maps were generated using the geopandas package in Python [33].

## Results

### Infectious diseases cases

According to the Libyan legislation, NRSS must track infectious diseases that could spread and become an epidemiological threat, with specific emphasis on certain diseases, as listed in the legislation. During this period, fifteen infectious diseases were reported with at least one case each; recorded data included the number of identified cases, the geographical distribution, and age group (<5 or ≥5 years) over the period of three months. The number of deaths was also reported. The highest number of cases reported was due to respiratory infection (14,031), followed by diarrhea, which was detected in 12,082 cases in the Northeastern Region of Libya. However, the infections reported least frequently were Measles and Bilharzia, with one case each, followed by malaria (5 cases). The case counts of rickettsiosis, leishmaniasis, and acute flaccid paralysis were reported at seven cases each. Other diseases were reported in descending order: pneumonia (655), rash without fever (174), chickenpox (170), rash with fever (153), hepatitis A (45), bloody diarrhea (20), and rubella (10). The highest number of respiratory infection cases was found in Benghazi (8,752 cases), while most diarrhea cases were reported from Albayda (7,475 cases). Three deaths were recorded: two due to diarrhea (case fatality rate [CFR]: 0.17/1,000) and one due to pneumonia (CFR: 1.53/1,000). A more detailed breakdown can be found in Table 1. The full curated data is attached in S1 and S2 Tables.

**Table 1:** Total number of infectious diseases reported three months after the flood in major districts in Eastern Libya.

District	Disease							Total
	Chicken Pox	Diarrhea	Pneumonia	Rash with fever	Rash without fever	Respiratory Infection	Other	
Albayda	57	7,475	74	39	46	1,564	5	9,260
Almarj	7	266	7	5	7	591	6	889
Benghazi	97	2,744	12	82	42	8,752	66	11,795
Derna	0	1,379	475	22	79	1,757	18	3,730
Ejdabia	9	71	0	0	0	168	3	251
Tobroq	0	147	87	5	0	1,199	5	1,443
Total	170	12,082	655	153	174	14,031	103	27,368

### Incidence rate of infectious diseases cases

The active surveillance system identified 27,368 infectious disease cases after the tremendous flood. The most frequent type of infectious disease reported after the flood was respiratory infections (14,031 cases), with an incidence rate of 701.67 per 100,000 population. Diarrhea was the second most frequent condition (12,082 cases), where its incidence rate was 604.21 per 100,000 population. Other significant cases were pneumonia (655 cases, incidence rate: 32.76/100,000 population), skin rash without fever (174 cases, incidence rate: 8.7/100,000 population), chickenpox (170 cases, incidence rate: 8.5/100,000 population), and skin rash with fever (153 cases, incidence rate: 7.65/100,000 population). Table 2 shows further details of reported infectious diseases and their incidence.

**Table 2:** Total number of infectious diseases reported during the period of three months after the flood, the percentage of cases by infectious disease type, and the incidence rate per 100,000 population of each disease. The total population of Eastern Libya (n = 1,999,646) served as the denominator for these calculations.

Disease	Cases	Deaths	% of All	Incidence Rate
Bilharzia	1	0	0	0.05
Bloody diarrhea	20	0	0.07	1.00
Chicken Pox	170	0	0.62	8.50
Diarrhea	12,082	2	44.15	604.21
Flaccid Paralysis	7	0	0.03	0.35
Hepatitis A	45	0	0.16	2.25
Leishmaniasis	7	0	0.03	0.35
Malaria	5	0	0.02	0.25
Measles	1	0	0	0.05
Pneumonia	655	1	2.39	32.76
Rash with fever	153	0	0.56	7.65
Rash without fever	174	0	0.64	8.70
Respiratory infection	14,031	0	51.27	701.67
Rickettsia	7	0	0.03	0.35
Rubella	10	0	0.04	0.50
**Total**	**27,368**	**3**	**100**	–

### Age group of infectious diseases cases

**Fig 1:**
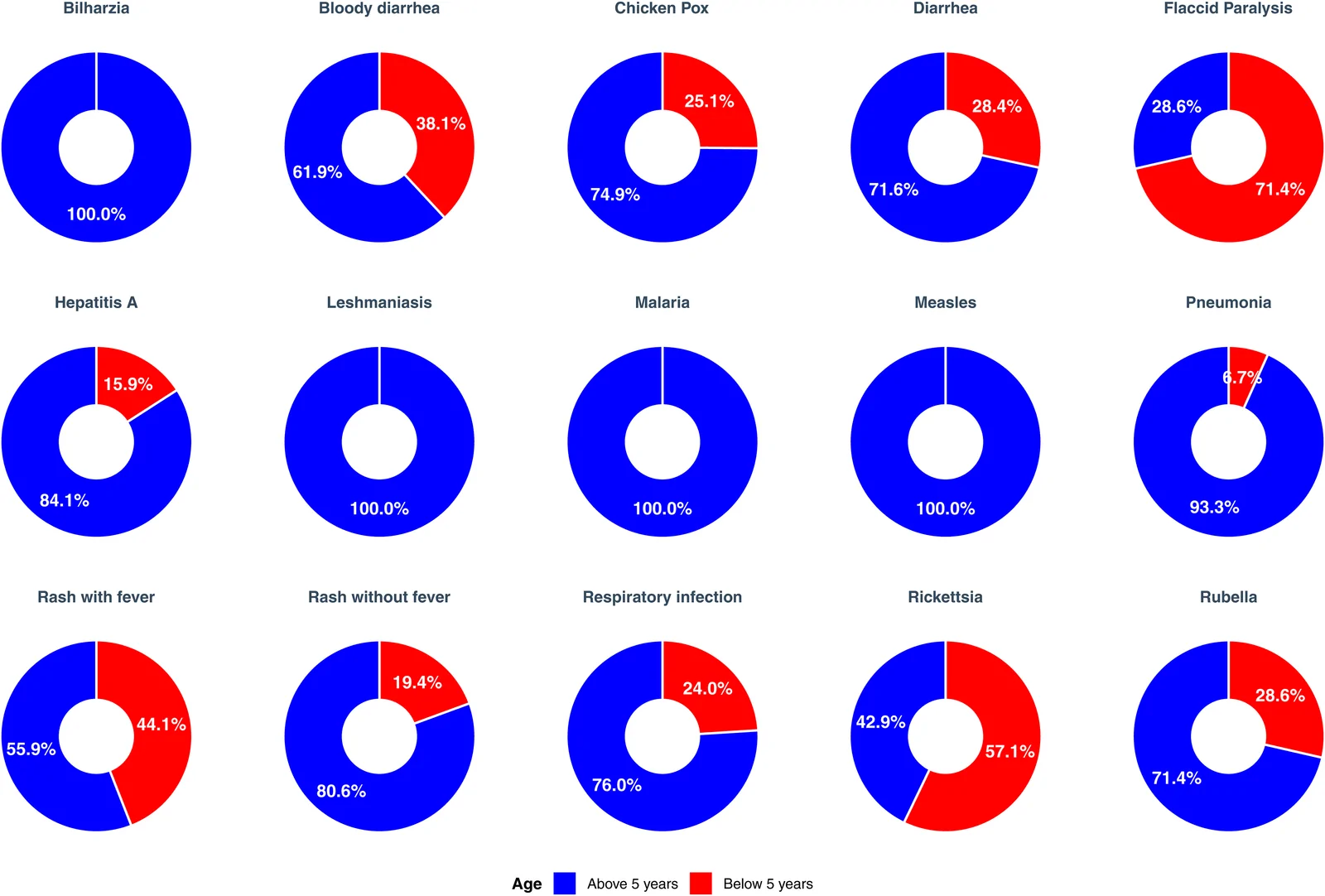
Percentage of age group under five (<5) and five years and older (≥5) for each reported infectious disease. Total population of children under five, at the time of the analysis, was recorded at 788,230, which represents 10.9% of the total Libyan population [32].

The incidence of rickettsiosis and acute flaccid paralysis (AFP) was higher in children under five years of age (<5), while all other diseases were more prevalent in the group aged five years and older (≥5). Bilharzia, measles, malaria, and cutaneous leishmaniasis were reported only with the ≥5 age group (Fig 1).

### Trend of diarrhea and respiratory infection cases

The number of diarrhea cases increased steadily until day 42 after the flood, when case counts peaked and then subsequently declined. Given the very large number of diarrhea cases, an urgent intervention was needed. Then, an educational intervention was implemented to raise awareness about using bottled water instead of groundwater in Albayda city, which had the highest number of diarrhea cases in the east. The result of the intervention was considerably noticeable as the number of diarrhea cases decreased in the final month of surveillance.

**Fig 2:**
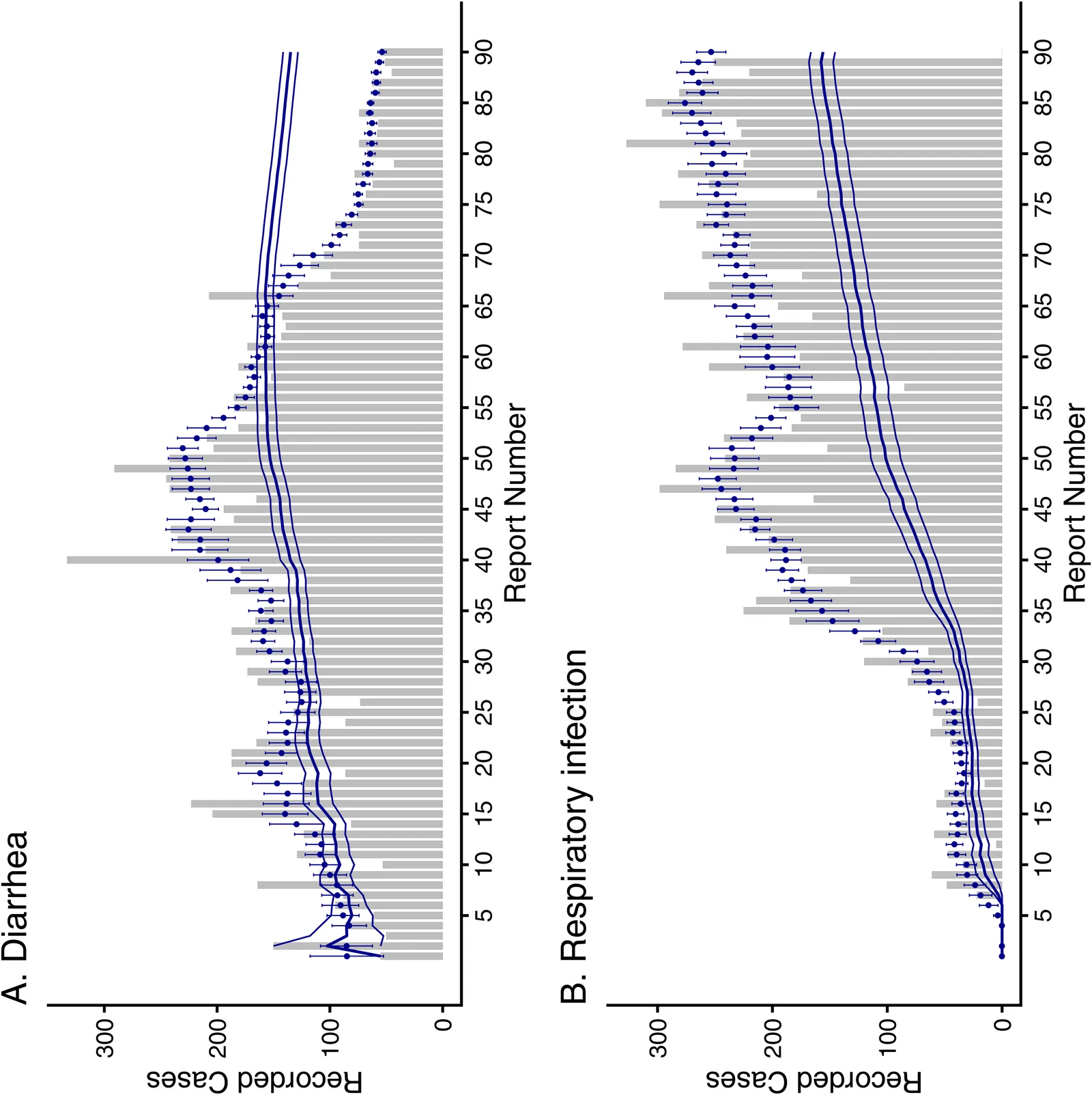
Temporal trend of diarrheal (A) and respiratory infection (B) cases, bars show the number of recorded cases corresponding to the report number, blue dots and error bars show a one-week moving average of new recorded cases and the standard deviation. The solid blue line represents the cumulative average with standard error (SE) margins.

**Fig 3:**
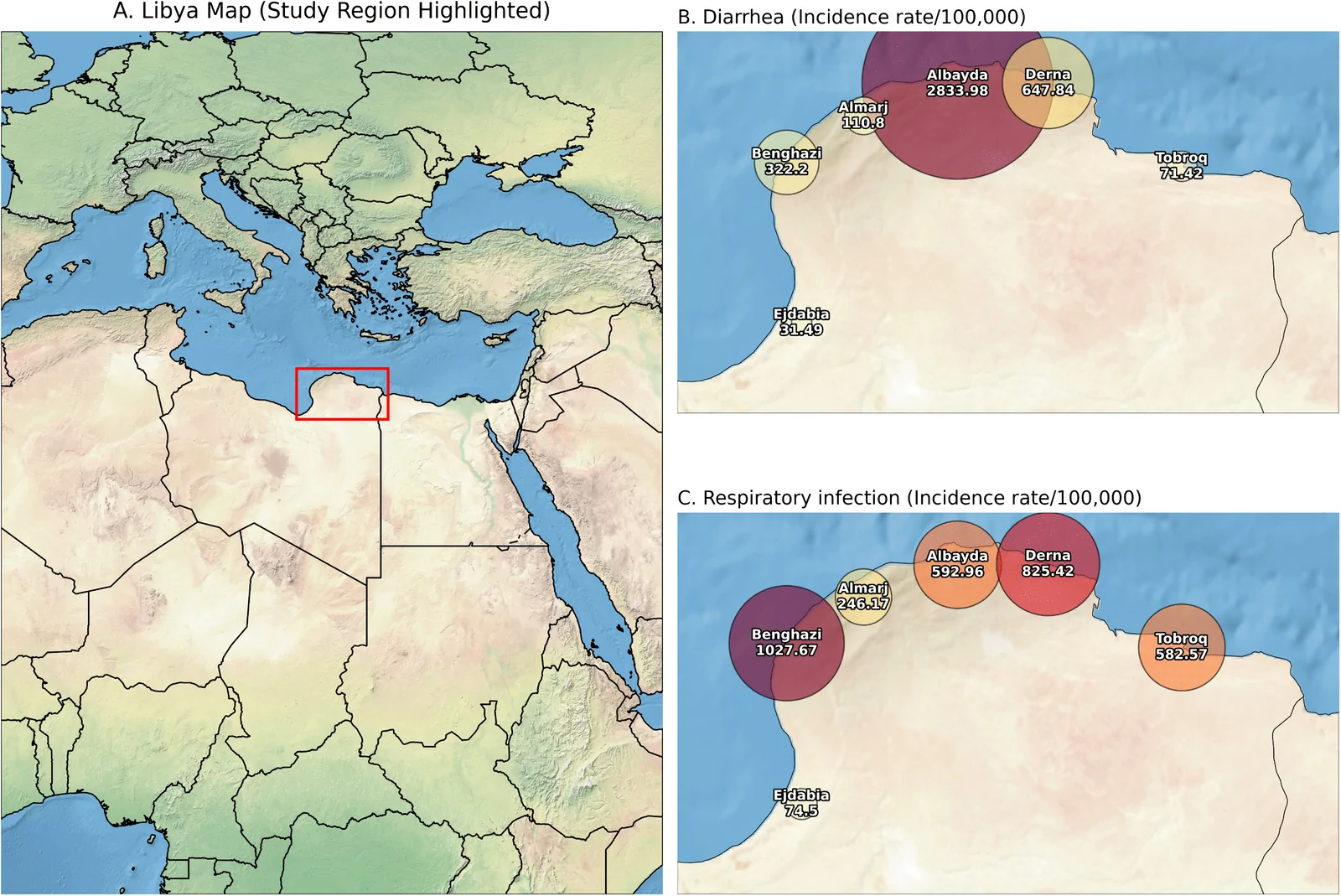
Geographical distribution of the district incidence rate for diarrhea (B) and respiratory infections (C) during the three-month period post-flood. The size and the color of the circles represent the magnitude of the incidence rate. The base shapefile for country boundaries and the background raster image were obtained from Natural Earth (https://www.naturalearthdata.com/downloads/10m-cultural-vectors/10m-admin-0-countries/) and (https://www.naturalearthdata.com/downloads/10m-raster-data/10m-natural-earth-1/), respectively.

Respiratory infection cases showed a steady increase over the total period, with a prominent increase after 30 days. The trend of increased incidence did not change for the whole 3 months of surveillance. Fig 2 summarizes the time trend of diarrheal (A) and respiratory infection (B) cases. Additionally, Fig 3 shows the geographical distribution of diarrheal (B) and respiratory infection (C) cases as well.

## Discussion

This study provides the first systematic summary of infectious disease surveillance following the 2023 floods in eastern Libya. Active surveillance of infectious diseases was conducted through the NRSS for three months (September 14 – December 24, 2023) after the disaster, issuing daily epidemiological reports.

The results of this study indicate a notable increase in communicable diseases, especially diarrhea and respiratory infections. The increase in diarrhea can be explained by disruptions in the sewage system, lack of clean water supply, and contamination of underground water tanks with overflowing sewage and compromised hygiene infrastructure. Furthermore, these environmental hazards may have impacted agricultural irrigation, thereby increasing the risk of food-borne disease transmission through contaminated produce.

The diarrheal cases peaked during the period from 40 to 50 days from the beginning of the flood. Subsequently, the number of reported cases declined until the end of the special active surveillance system recording in the affected area. This trend can be attributed to several factors. One reason is that the depletion of emergency water supplies, caused by declining political and media attention and/or the exhaustion of local reserves, forced people to return to using previously contaminated water sources, such as household water tanks or underground wells. The reports indicate that people were still using underground water for washing and cleaning [34] likely due to insufficient public health education regarding the risks of using untreated groundwater. Previous studies have shown that this critical period can extend up to seven weeks, which is consistent with our findings [35]. Additionally, the incubation period of the causative organisms could be another reason. Among the mitigation strategies that followed and had a significant impact were the provision of clean and bottled water, and public health education campaigns [36,37]. Such strategies are likely to have contributed to the reduction in cases following their initial peak.

Even though Derna suffered most of the impact due to the dams’ collapse, Albayda recorded the highest rainfall, reaching 414 mm, contrasting with 100 mm in Derna [38] and therefore, also experienced a great detrimental impact from the storm. Albayda is the second-largest city in the area, with a major hospital serving an extensive geographical region, and reported the highest number of diarrhea cases. Examining the drinking water from local wells in Albayda revealed contamination with *Escherichia coli* species [36].

Despite the absence of reported cholera cases [36,39], other enteric pathogens such as *Entamoeba histolytica*, *Entamoeba coli*, and *Escherichia coli* were detected [39]. This is consistent with a study done in China after a cyclone showing no increase in bacillary dysentery, cholera, typhoid, or paratyphoid fever, but where infectious disease cases related to other causes were significantly increased. *Escherichia coli* was listed as one of these causes, in line with our observation [40].

The increase in respiratory infections is likely attributable to the large-scale displacement of populations (IDPs; Internally Displaced Person) [41]. The number of IDPs after the flood was more than 44,000 individuals in October 2023 [42]. Furthermore, the implementation of the active surveillance system likely contributed to this trend as it captured routine seasonal respiratory illnesses in addition to outbreak-related cases.

The continued increase in respiratory infection cases throughout the surveillance period, in contrast to diarrhea, which declined over time, may be attributed to the prolonged nature of internal displacement (IDP), which typically requires longer-term resolution

From a public health perspective, the findings reveal significant gaps in health education regarding the safe use of clean water for drinking, cooking, and washing. This is supported by the large number of diarrheal cases reported in Albayda, where underground water was contaminated with *Escherichia coli* [43]. The data suggest that the population was not adequately warned against using underground water, despite a delayed awareness campaign mentioned in Report 90 [39].

The surge in cases was likely aggravated by the absence of an urgent early educational campaign at the onset of the disaster, particularly about avoiding contaminated water sources. These observations underline the urgent need for better education planning and preparedness. In addition to education, repairing and assessing the sewage system is essential to prevent infiltration or direct discharge into underground water sources [44]. Additionally, socio-economic constraints could be another factor to exacerbate the situation, as residents were likely not able to utilize clean water for cooking and washing. A similar observation was reported in Pakistan [45].

Among other expected health-related hazards after the catastrophic flood was the unsanitary shelter of IDPs, contaminated food, dead body odour, crowding, stagnant water, and lack of local health services. Such hazards not only compromise physical health but may further impact the psychosocial well-being of residents in the area by exacerbating their mental status, potentially leading to anxiety and depression, especially among IDPs [13].

This study constitutes a message to public health officers regarding the importance of continuing the active surveillance system beyond the first few weeks after a disaster, as supported by the peak in diarrheal cases observed. A further important message is the necessity of ensuring fair distribution of health post-disaster services, which should be informed by data and statistics rather than geographical proximity to the disaster location or disproportionate media attention.

There are four main limitations of this paper. First, Reports after weekends may show an abrupt increase in the number of new cases due to the aggregation of multi-day case counts. This limitation was mitigated by using a moving average as mentioned previously. Secondly, there is no clear recording of the interventions that happened to find out their impact on the disease incidence. However, this paper is interested in describing the epidemiological status of the disease more than the impact of applied interventions. Thirdly, published baseline information on the incidence of infectious diseases is scarce, which makes the comparison to the baseline impossible. Furthermore, the routine surveillance system is predominantly passive, in contrast to the active surveillance implemented during this emergency period, which further complicates the comparison process. Lastly, there is a possibility of under- or inappropriate reporting of cases, due to the geographical widespread effect of the disaster and the acute onset of the flooding. The exclusion of the private healthcare sector from the active surveillance system may also lead to missed cases.

In conclusion, this study is the first comprehensive report on the communicable disease situation following the 2023 Libyan floods. It revealed an increased incidence of diarrheal and respiratory diseases and highlighted possible risk factors contributing to this rise. These findings call for urgent intervention and strategic planning, particularly in public health education, emergency preparedness (specifically regarding potable water security), and infrastructure improvement. These measures should help Libyan cities better withstand future disasters.

## Supporting information

S1 Table Full table of recorded cases. (PDF)

S2 Table The raw data curated from the reports. (XLSX)
