# Supplementary material for: Post-flood communicable disease surveillance following the 2023 dam collapse in Eastern Libya
Source: PLOS Glob Public Health. 2026 Aug 20;6(8):e0007124. doi: 10.1371/journal.pgph.0007124 (PMC13492783; doi:10.1371/journal.pgph.0007124)
Supplement: Supplementary file 1 [file pgph.0007124.s001.pdf]

Location	Population	Bilharzia	Bloody diarrhea	Chicken Pox	Diarrhea	Flaccid Paralysis	Hepatitis A	Leshman-iasis	Malaria	Measles	Pneumonia	Rash with fever	Rash w/o fever	Resp. infection	Rickettsia	Rubella	Total
Albayda	263763	0 (0.00)	2 (0.76)	57 (21.61)	7475 (2,833.98)	0 (0.00)	1 (0.38)	0 (0.00)	0 (0.00)	1 (0.38)	74 (28.06)	39 (14.79)	46 (17.44)	1564 (592.96)	1 (0.38)	0 (0.00)	9260
Almarj	240076	0 (0.00)	0 (0.00)	7 (2.92)	266 (110.80)	0 (0.00)	0 (0.00)	0 (0.00)	0 (0.00)	0 (0.00)	7 (2.92)	5 (2.08)	7 (2.92)	591 (246.17)	5 (2.08)	1 (0.42)	889
Benghazi	851637	0 (0.00)	10 (1.17)	97 (11.39)	2744 (322.20)	7 (0.82)	39 (4.58)	1 (0.12)	5 (0.59)	0 (0.00)	12 (1.41)	82 (9.63)	42 (4.93)	8752 (1,027.67)	1 (0.12)	3 (0.35)	11795
Darnah	212861	1 (0.47)	5 (2.35)	0 (0.00)	1379 (647.84)	0 (0.00)	0 (0.00)	6 (2.82)	0 (0.00)	0 (0.00)	475 (223.15)	22 (10.34)	79 (37.11)	1757 (825.42)	0 (0.00)	6 (2.82)	3730
Ejdabia	225496	0 (0.00)	0 (0.00)	9 (3.99)	71 (31.49)	0 (0.00)	3 (1.33)	0 (0.00)	0 (0.00)	0 (0.00)	0 (0.00)	0 (0.00)	0 (0.00)	168 (74.50)	0 (0.00)	0 (0.00)	251
Tobroq	205813	0 (0.00)	3 (1.46)	0 (0.00)	147 (71.42)	0 (0.00)	2 (0.97)	0 (0.00)	0 (0.00)	0 (0.00)	87 (42.27)	5 (2.43)	0 (0.00)	1199 (582.57)	0 (0.00)	0 (0.00)	1443
Total	1999646	1 (0.05)	20 (1.00)	170 (8.50)	12082 (604.21)	7 (0.35)	45 (2.25)	7 (0.35)	5 (0.25)	1 (0.05)	655 (32.76)	153 (7.65)	174 (8.70)	14031 (701.67)	7 (0.35)	10 (0.50)	27368

Supplementary Table 1: This table shows the cumulative number of case counts and corresponding incidence rates of reported diseases across all affected districts. The upper values represent the case counts, while the figures in parentheses indicate the incidence rate per 100,000 population.
