# Supplementary material for: Post-flood communicable disease surveillance following the 2023 dam collapse in Eastern Libya
Source: PLOS Glob Public Health. 2026 Aug 20;6(8):e0007124. doi: 10.1371/journal.pgph.0007124 (PMC13492783; doi:10.1371/journal.pgph.0007124)
Supplement: Supplementary file 2 [file pgph.0007124.s003.pdf]

Dear Editor and Reviewers,

We have gratefully received your comments and suggestions to improve our manuscript. We considered each suggestion carefully and followed them. Those comments were very helpful for us to improve the quality of our work.

Moreover, to improve the manuscript's English, we asked our colleague, Dr. Hager Kowash, a native English speaker, to help with proofreading and final manuscript preparation. We believe the language level is much better now. We did our best to fulfill this requirement as shown in the revised manuscript.

We carefully followed the guidelines of PLOS Global Public Health and created the required files and figures according to the requirements. We have prepared a point-to-point answer here in this "Response to Reviewers" document. We hope these changes make our manuscript suitable for publication.

Journal Requirements:

Comment:

1. We ask that a manuscript source file is provided at Revision. Please upload your manuscript file as a .doc, .docx, .rtf or .tex.

Response:

We are glad to follow your guidelines by uploading the source file of our manuscript as one clean TeX file, including reference information.

Comment:

2. Please upload separate figure files in .tif or .eps format. Also, remove the figures from your manuscript file but keep the legends.

   For more information about figure files please see our guidelines:

   `https://journals.plos.org/globalpublichealth/s/figures`

   `https://journals.plos.org/globalpublichealth/s/figures#loc-file-requirements`

Response:

We prepared the figures in the format required, and uploaded them according to this comment.

Comment:

3. We note that your Data Availability Statement is currently as follows: Data is provided in the manuscript, further inqueries will be considered by the authors.

   Please confirm at this time whether or not your submission contains all raw data required to replicate the results of your study. Authors must share the "minimal data set" for their submission. PLOS defines the minimal data set to consist of the data required to replicate all study findings reported in the article, as well as related metadata and methods

   `http://journals.plos.org/globalpublichealth/s/data-availability#loc-minimal-data-set-definition`

   For example, authors should submit the following data:

   - The values behind the means, standard deviations and other measures reported;

   - The values used to build graphs;

   - The points extracted from images for analysis.

   Authors do not need to submit their entire data set if only a portion of the data was used in the reported study.

If your submission does not contain these data, please either upload them as Supporting Information files or deposit them to a stable, public repository and provide us with the relevant URLs, DOIs, or accession numbers. For a list of recommended repositories, please see

`http://journals.plos.org/globalpublichealth/s/recommended-repositories`

If there are ethical or legal restrictions on sharing a de-identified data set, please explain them in detail (e.g., data contain potentially sensitive information, data are owned by a third-party organization, etc.) and who has imposed them (e.g., an ethics committee). Please also provide contact information for a data access committee, ethics committee, or other institutional body to which data requests may be sent. If data are owned by a third party, please indicate how others may request data access.

Response:

The full data is uploaded in the supplementary section as EXCEL file. And the data availability statement was modified accordingly to "All data is available in the manuscript and its attached supplementary materials"

Comment:

4. Some material included in your submission may be copyrighted. According to PLOS's copyright policy, authors who use figures or other material (e.g., graphics, clipart, maps) from another author or copyright holder must demonstrate or obtain permission to publish this material under the Creative Commons Attribution 4.0 International (CC BY 4.0) License used by PLOS journals. Please closely review the details of PLOS's copyright requirements here: PLOS Licenses and Copyright. If you need to request permissions from a copyright holder, you may use PLOS's Copyright Content Permission form.

   Please respond directly to this email or email the journal office and provide any known details concerning your material's license terms and permissions required for reuse, even if you have not yet obtained copyright permissions or are unsure of your material's copyright compatibility.

Response:

We assure the esteemed editorial team that no copyrighted materials were used in the preparation of this manuscript, with the exception of the map shapefile and the raster file (used to enhance the map's visual quality). Both files were obtained from Natural Earth, which provides its data in the public domain (CC0). Therefore, no specific permission is required for their use or publication.

here are the links:

– shape file: `https://www.naturalearthdata.com/downloads/10m-cultural-vectors/10m-admin-0-countries/`,

– and here is the link to the raster file: `https://www.naturalearthdata.com/download/10m/raster/NE1_HR_LC_SR_W.zip`

Potential Copyright Issues:

Comment:

a. Figure 3: please (a) provide a direct link to the base layer of the map (i.e., the country or region border shape) and ensure this is also included in the figure legend; and (b) provide a link to the terms of use / license information for the base layer image or shapefile. We cannot publish proprietary or copyrighted maps (e.g. Google Maps, Mapquest) and the terms of use for your map base layer must be compatible with our CC-BY 4.0 license.

Note: if you created the map in a software program like R or ArcGIS, please locate and indicate the source of the basemap shapefile onto which data has been plotted.

If your map was obtained from a copyrighted source please amend the figure so that the base map used is from an openly available source. Alternatively, please provide explicit written permission from the copyright holder granting you the right to publish the material under our CC-BY 4.0 license.

Please note that the following CC BY licenses are compatible with PLOS license: CC BY 4.0, CC BY 2.0 and CC BY 3.0, meanwhile such licenses as CC BY-ND 3.0 and others are not compatible due to additional restrictions.

If you are unsure whether you can use a map or not, please do reach out and we will be able to help you. The following websites are good examples of where you can source open access or public domain maps:

* U.S. Geological Survey (USGS) - All maps are in the public domain. (`http://www.usgs.gov`)
* PlaniGlobe - All maps are published under a Creative Commons license so please cite "PlaniGlobe, `http://www.planiglobe.com`, CC BY 2.0" in the image credit after the caption. (`http://www.planiglobe.com/?lang=enl`)
* Natural Earth - All maps are public domain. (`http://www.naturalearthdata.com/about/terms-of-use/`)

Response:

The map data is obtained from Natural Earth, which means there is no permission is required to use it.

The shape file: `https://www.naturalearthdata.com/downloads/10m-cultural-vectors/10m-admin-0-countries/`

raster file: `https://www.naturalearthdata.com/downloads/10m-raster-data/10m-natural-earth-1/`

Additional Reviewer comments

Reviewer #1

Abstract

Comment:

The authors have mentioned only two types of infectious diseases. What about the others?

Response:

We would like to thank the reviewer for the time and effort spent. The suggestions are useful and helped us improve our manuscript.

We have updated the section of results in the abstract to include more information related to other diseases such as chickenpox and measles. We highlighted that Cholera was not detected, which is a fundamental finding in our paper.

Comment:

The conclusion needs to be accurate and should clearly reflect the study findings.

Response:

We updated the conclusion, removed the first sentence, and added the main important findings and recommendations of the paper according to the reviewer's suggestions.

Comment:

Keywords are missing.

Response:

Keywords are added at the end of the abstract.

Introduction

Comment:

The introduction requires a more comprehensive and strengthened literature review relevant to the study.

Response:

The introduction is extensively enriched regarding previous study observations and litratue review as demonstrated in the paper.

Comment:

The impact of floods on infectious diseases and mitigation strategies is missing.

Response:

This is already mentioned in the second paragraph of the Introduction, mentioning important factors: disrupted sanitation, contaminated water sources, and population displacement, which affect the spread of infectious diseases.

However, we appreciate the reviewer comment and subsequently we have updated the introduction to cover this more extensively including information from other areas, such as Asia, Latin America, and Sub-Saharan Africa.

Regarding mitigation: we followed the reviewer comment and addressed that in the discussion section.

Comment:

The authors discuss flood-related damages from 2024, while the study focuses on infectious disease events during the 2023 floods.

Response:

This analysis is about the worldwide impact of floods to show the importance of having reports of the cases affected and to analyze the performance of the surveillance system. To increase the details, we revised the statement to include the major impact of floods over the years from 2020 to 2025.

Comment:

The need for the study and its novelty should be clearly stated.

Response:

Thank you for raising this point, it was a clear shortage of the introduction. We have followed the reviewer's comment and added two paragraphs to address this point at the end of the introduction.

Methods

Comment:

The methods section is insufficient and should be more comprehensive.

Response:

We agree with the reviewer's point and subsequently revised the methods section extensively and added more details.

Comment:

The criteria for including reports need to be clearly described.

Response:

All reports were included in the analysis process. We added one sentence to clarify that in the methodology section.

Comment:

There is no information on how the validity of the reports was assessed. Are there any references for this?

Response:

Official reports were issued by the National Center for Disease Control (NCDC), in accordance with the regulatory framework of Libyan Health Code No. (106) of 1973. The surveillance data are collected by trained physicians and allied health professionals as part of an established reporting system, rather than through volunteer-based efforts. Due to the standardized nature and long-standing legal mandate of this national surveillance infrastructure, the data are considered valid. Subsequently, the authors did not consider further independent validation of the reporting system. Clarification was added in the paper text.

Comment:

Which surveillance method was used in this study?

Response:

The regular surveillance system is passive during routine surveillance, but in case of an outbreak and/or disaster, the system becomes active, as in this study. This involves daily fieldwork, including visits to health facilities and the direct collection of data from relevant emergency departments. The raw data collected are then transmitted to the national level at around 10:00 pm; the results are subsequently reported at midnight of the same day in an official report published to all related members and the stakeholders, such as WHO and UNICEF.

Comment:

There is no data provided on the fourteen diseases. How were the cases defined, and what were the criteria for selecting these diseases?

Response:

This is regulated by the Libyan Law 106: 1973 about the health regulation. We added clarification for this point in the paper and cited the law in the references. It is noteworthy that the law mandates the reporting of any infectious disease case detected.

Comment:

How were the incidence rates of the diseases calculated?

Response:

The incidence rate was calculated by dividing the number of reported infectious disease cases by the total population-at-risk within the flood-affected municipalities of eastern Libya. This denominator was derived from the most recent 2023 Bureau of Statistics and Census (BSC) estimates. We added this information in the manuscript.

Results

Comment:

The text should clearly refer to the tables.

Response:

We carefully followed this comment, now the two tables are referred to in the text.

Comment:

All fourteen listed diseases should be mentioned in the text, along with their numbers or percentages.

Response:

Thank you very much, we followed the comment and added all diseases, we also included all disesease even with only one case. first time measles and bilharzia was omitted because of only one case of each were reported.

Comment:

The authors should also report case fatality rates and prevalence per 1,000 population in addition to incidence rates.

Response:

The number of deaths reported related to infectious diseases were 3 cases (2 because of Diarrhea (0.17/1000) and 1 because of Pneumonia (1.53/1000). We added this to the discussion section.

Comment:

Age groups have been divided into less than and more than five years. What about adults, older adults, and pregnant and lactating women (PLWs)?

Response:

The system follows the WHO classification of cases for more than five years and less than five years only. Reports did not mention any other age groups.

Comment:

Why did the authors focus only on the trend of diarrhea? What about trends in other diseases? Which type of analysis was used to assess these trends?

Response:

The trends of all diseases were analyzed. This was done by calculating the accumulated mean of the data, and the one-week moving average to smoothen the output and reduce the effect of daily variation. Diarrhea was selected because it was the only disease that showed a significant and interesting trend. However, after this comment, we added the respiratory infection trend also. The case numbers of other diseases are small, which makes the trend not informative.

Discussion

Comment:

The discussion should be structured and developed based on the points raised above.

Response:

We have revised the discussion section thoroughly following the points raised above.

- We changed the flow of the writing in the discussion section to make it more fluent and smooth.
- We added more information according to the points raised by the reviewers.
- We extensively revised the English language and improved it substantially

Reviewer #2

Comment:

Review of “Post-Flood Communicable Disease Surveillance Following the 2023 Dam Collapse in Eastern Libya. “

To the Authors,

The manuscript “Post-Flood Communicable Disease Surveillance Following the 2023 Dam Collapse in Eastern Libya” provides new information about the health impacts of the 2023 flood and dam collapses in Libya. Because the global climate is warming, more cyclones are expected in the future, and this study provides very useful information for those medical and public health officials who will manage future disasters. The availability of active surveillance data make this study possible, and the authors described the outbreak well in the tables and figures. Several recommendations follow to further strengthen the manuscript.

Response:

Thank you very much to the reviewer for spending time and effort reading the paper, and the valuable comments he/she provided which we used to improve our manuscript.

Comment:

Major

Context about the collapse of the dams is lacking in the manuscript. A brief description and the findings of Nemnem et al 2025 “How extreme rainfall and failing dams unleashed the Derna flood disaster” (10.1038/s41467-025-59261-9) in Nature Communications would give readers important background information and should be added to the manuscript.

Response:

Thank you very much for suggesting this incredible article. Eventhough the content of that article is more related to technical issues of dam safety and structural stability, we incorporated few ideas from it in the introduction.

Comment:

More information in the Introduction section about the public health response would be very useful. Were handwashing facilities made available? Latrines? Soap? Methods for point-of-use water disinfection?

Response:

Unfortunately, no such information made availble by the reports we examined.

Comment:

Line 48: Written authorization was obtained. Please clarify – was an ethical review part of the process for authorization?

Response:

This study is based on the analysis of publicly available, aggregated daily reports issued by the National Center for Disease Control (NCDC) to the general public and health stakeholders. We have no access to individual patient records, clinical samples, or any personally identifiable information (PII), this work is classified as non-human subjects research. Under standard ethical protocols (such as those outlined in the Declaration of Helsinki), formal IRB/ethical approval is not required for the secondary analysis of anonymous, public-domain aggregate statistics. We have updated the Ethical Considerations statement in the methodology section to reflect this status more accurately.

Comment:

Data generated by the active surveillance system is very important. Equally important would be a clear description of the how the active surveillance system worked. In the Discussion section, the manuscript notes "NRSS depends on official public health officers and doctors to report data from all 94 hospitals and health centers that have the ability to provide emergency health services." This section should be moved the end of the first paragraph of the Methodology section. Additional information should be added about whether the system is paper-based or electronic and whether "health centers" includes (private) physician offices or whether the system only includes public (governmental) health centers.

Response:

- We have moved this paragraph mentioned in the comment according to the reviewer's suggestion.
- We added more details regarding to the surveillance system in the paper following this comment.

Comment:

If private physician offices do not submit reports through the NRSS, then the Discussion section should note this as a reason to believe the reported case numbers are an underestimate of actual cases of disease.

Response:

It was added in the limitation part of the discussion.

Comment:

Is any information available about the number of people hospitalized due to diarrhea, pneumonia, or respiratory illness? Is any information available about the number people seen in emergency departments due to diarrhea, pneumonia, or respiratory illness? If

so, that information should be added to the Results section.

Response:

Unfortunately, no information available about the hospitalization, or any other information other than reported in the NRSS reports.

Comment:

The active surveillance system began collecting data only after the flood and dam collapse were not available. The discussion section could note that no “pre-collapase” and “post-collapse” comparisons are possible. However, pre-collapse, there were also cases of diarrhea and respiratory symptoms. Thus, it should be noted in the Discussion section that no “baseline” data are available for comparison.

Response:

Added to the limitation section.

Comment:

The Discussion section could be strengthened by adding several sentences about lessons learned to guide public health professionals who will manage future floods elsewhere.

Response:

Thank you for this valuable suggestion, we have followed it and added a few sentences to the discussion accordingly.

Comment:

Other comments

Lines 5-8: “Like many Mediterranean and North African countries, Libya is increasingly vulnerable to climate-related extreme events due to aging infrastructure and limited early-warning capacity. Living in flood-prone areas—especially where early warning systems and flood-related education are lacking—greatly increases the risk of flood-related harm [1, 3].”

The text before this provided a general view of flooding and so does the text that follows in line 8-9, so this information specific to Libya is out of place. It would fit better just before line 23: “In Libya, on 10 September 2023, heavy rainfall and strong winds from Tropical Storm Daniel caused the collapse of two dams,”

Response:

Done

Comment:

Lines 8-9: “In 2024, floods caused 5,885 deaths, affected 48 million people, and cost an estimated 32 billion USD [4].” Make it clear that these number are global, not specific to the collapse of the Derna dams.

Response:

Done

Comment:

Line 51: spell out BSC.

Response:

Done

Comment:

Line 58: “diarrhea” instead of “Diarrhea”

Response:

Done

Comment:

Line 59: Instead of “However, the lowest cases were rubella,. . . .” change to “The infections reported least frequently were rubella. . . ”

Response:

Done

Comment:

Line 64: The term disease burden generally refers to the combination of disease frequency and disease severity. Because severity data are not available, the text should be changed from “The biggest burden of the infectious disease after the flood. . . ” to “The most frequent type of infectious disease reported after the flood. . . .”

Response:

Done

Comment:

Line 78=79: Instead of “Diarrhea cases were significantly increased in each day, reaching the peak in day of 42. . . ” change to “The number of diarrhea cases increased steadily

until day 42 when case counts peaked and then declined."

Response:

Done

Comment:

Lines 79-80: "The interpretation of the diarrhea situation was decided as an outbreak which needs an urgent intervention." Change to "Given the very large number of diarrhea cases, an urgent intervention was needed."

Response:

Done

Comment:

Lines 81-82 ": .. groundwater in Albayda city, which suffers from the highest number of diarrhea cases in the east." Change to "groundwater in Albayda city, which experienced the highest number of diarrhea cases in the east."

Response:

Done

Comment:

Lines 103-104: "This contamination may also have affected crop growing, increasing the risk of further infection spreading." Change to "This may have had contaminated food crops, increase the risk of food-borne disease transmission."

Response:

Done

Comment:

Line 109: Were the "underground wells" dug (shallow) wells or deeper drilled well (or "tube wells")?

Response:

There is no reference to this information in the reports published.

Comment:

Line 118: Change "Cholera" to "cholera" (the disease is not capitalized).

Response:

Done

Comment:

Line 119-120: “This is going well with another survey conducted in China showing no increase in Bacillus Dysentery, Cholera. . . ” The comparison to the pathogens identified in China after a cyclone is not needed and can be deleted. If the authors want to keep this sentence, change the beginning to “This is consistent with a study done in China after a cyclone. . . ”

Response:

Done

Comment:

Line 123: Change “Respiratory infections are probably due to IDP. . . ” to “Crowding due to the large number of internally displaced persons (IDP) probably promoted the transmission of respiratory infections.”

Response:

Done

Comment:

Lines 138-140: Please add information about whether the response included the provision of liquid chlorine (hypochlorite solution) for point-of-use drinking water disinfection.

Response:

There is no infomration in the original reports about using liquid chlorine to disinfect drinking water.

Comment:

Line 147-148: Add mental health conditions such as anxiety and depression to the list of problems suffered by IDP.

Response:

Done, reference added

Reviewer #3

Comment:

Reviewer #3: Thank you for bringing to light this evidence and for all of your work writing this piece. I was excited to read the piece, and I think a comparison to a similar season in a prior year could be a really interesting piece.

Here are some of my more detailed comments. Libya dam collapse article:

Response:

We appreciate the reviewer suggestions and time spent to read and comment on our manuscript, without this help, the paper won't be improved.

Regarding the baseline incidence, our internal records show that during the first half of 2023, there were only 1,306 reported cases of diarrhea in the study area. This is too small compared to the nearly 12,000 cases observed in the three months following the flood. However, as we do not possess comparative surveillance data for the corresponding season (September–December) of 2022 or 2024, we chose not to include this comparison in the manuscript to avoid potential seasonal bias. Moreover, the type of surveillance is different post-flood (active) from the usual (passive). We believe focusing on the post-flood trend provides a more scientific analysis, given the available information.

To improve transparency, we added this to the limitations of the study

Comment:

Abstract: led not leading

Response:

Done

Comment:

Would love to see a sentence in abstract about implications of this study be more applicable than just to lybia — what does this study say or contribute to in regards to literature about impacts of floods.

Response:

This study does not only reflect the impact of flooding in Libya alone, but it also extends to other countries with similar situations of limited resources and preparedness. That is supported by the scarcity of published of similar articles in countries of North Africa for example.

We added one sentence to the abstract to reflect this idea.

Comment:

Line 1: How do you define most frequent. I think this phrase could be deleted

Response:

Removed

Comment:

Line 8: in which geographic region?

Response:

This information is worldwide, We updated the text to show that.

Comment:

Line 16: worsened is not right word to put here

Response:

Replaced with “substandard”

Comment:

Line 21: Citation needed

Response:

Another reference was added.

Comment:

Line 25: cities in the northeast of Libya

Response:

Modified.

Comment:

Line 27: of which nation?

Response:

Libyan, it is added to the manuscript for clarification.

Comment:

Could present incidence rate per 100,000 instead of million to make more aligned with WHO reports

Response:

We have updated the tables and the number in text to follow this comment.

Comment:

Fig 1: I have hard time interpreting because I do not know what % of the population is under 5. So do not know what is similar to population structure and what is disproportionately affecting kids <5

Response:

According to libyan BSC in 2023, 10.9% of the total population are under 5 years old, corresponding to approximately 230,930 in the study area. We have made it clear in Fig. 1 caption.

Comment:

I think you should compare the rates of all these diseases to a year before the flood and conduct using a difference in difference analysis in order to make a causal claim.

I have no way of knowing in the paper right now if any of these are different than the normal rates in these cities for this time of year.

Response:

There is no such information available; we added this to the limitations. For that reason, this information gap enhances the importance of this article as a base line information for such events that can be used for comparison in the future.

Comment:

Fig 3 I do not know why the faceting is the way it is. Can you show the difference in incidence by city please instead of totals?

Response:

We have changed the presentation of the data to show the incidence in the tables, and Figure 3. To do that, we have to adopt districts instead of cities, because we have no detailed information about the population of each city. Following this comment, we revised the tables, figures, and text of the whole paper to be able to satisfy this very important suggestion.

Comment:

Figure Resubmissions:

While revising your submission, we strongly recommend that you use PLOS's NAAS tool (`https://ngplosjournals.pagemajik.ai/artanalysis`) to test your figure files. NAAS can convert your figure files to the TIFF file type and meet basic requirements (such as print size, resolution), or provide you with a report on issues that do not meet our requirements and that NAAS cannot fix.

After uploading your figures to PLOS's NAAS tool - `https://ngplosjournals.pagemajik.ai/artanalysis`, NAAS will process the files provided and display the results in the "Uploaded Files" section of the page as the processing is complete. If the uploaded figures meet our requirements (or NAAS is able to fix the files to meet our requirements), the figure will be marked as "fixed" above. If NAAS is unable to fix the files, a red "failed" label will appear above. When NAAS has confirmed that the figure files meet our requirements, please download the file via the download option, and include these NAAS processed figure files when submitting your revised manuscript.

Response:

Thank you again for your valuable feedback. We hope that the revised version of our manuscript will address all of your concerns and meet the standards for publication.
