# Supplementary material for: Post-flood communicable disease surveillance following the 2023 dam collapse in Eastern Libya
Source: PLOS Glob Public Health. 2026 Aug 20;6(8):e0007124. doi: 10.1371/journal.pgph.0007124 (PMC13492783; doi:10.1371/journal.pgph.0007124)
Supplement: Supplementary file 3 [file pgph.0007124.s004.pdf]

Review assignment: PGPH-D-25-03695R1 - [EMID:172d392a6ec7c678]

Overall Feedback
This manuscript addresses an important public health issue in the aftermath of the September 2023 floods in Libya. However, substantial revisions (major) are required before the manuscript can be considered for publication. In particular, the introduction and methods sections are currently too generic and would benefit from significant structural revision to better contextualise the study within Libya's unique environmental, infrastructural, and post-conflict setting, while providing greater methodological detail and transparency.

Abstract
The abstract could be strengthened by providing additional detail on the study design and quantitative methods employed. Rather than simply stating that the analysis was conducted in R, the authors should briefly describe the ecologic descriptive analytical approach used and the rationale behind it. This would provide readers with a clearer understanding of the study methodology and findings.

Introduction
Would it be appropriate to include Libya's current post-conflict situation as an additional and potentially unique risk factor contributing to the impacts of flood events? Sustained damage resulting from both internal and external conflicts has likely affected critical infrastructure and may have reduced the country's resilience to environmental disasters.

Although the manuscript discusses several broad risk factors for diarrhoeal disease, the discussion remains largely generic. This section would benefit from a stronger focus on Libya-specific determinants of disease transmission. For example, Libya's predominantly arid environment and associated water scarcity may increase reliance on alternative water sources, potentially elevating the risk of diarrhoeal infections. Furthermore, damage to sanitation and wastewater infrastructure during years of conflict may have increased population exposure to waterborne pathogens. Incorporating these contextual factors would help situate the study within the specific environmental and socio-political realities of Libya.

The introduction would also benefit from providing greater context regarding the September 10, 2023 flood disaster. While the event was triggered by an extreme weather event, it is important to discuss the broader factors that contributed to the catastrophe. For example, were the affected dams already structurally weakened due to conflict-related damage, inadequate maintenance, ageing infrastructure, or a combination of these factors? Such discussion would provide valuable context that is Libyan centred and help readers better understand why the disaster occurred. Additionally, the names and locations of the two dam structures involved in the disaster should be clearly stated.

Methods
The methods section would benefit from the inclusion of a dedicated Study Area subsection describing Libya's geographic, demographic, and socioeconomic characteristics. Such a section would help readers unfamiliar with the country

better understand the study context. I also strongly recommend including a map illustrating Libya's administrative boundaries, major population centres, the locations of the affected dams, and the relevant river or watershed systems associated with the flood event. This would substantially improve the geographical context of the study.

More broadly, the methods section is currently underdeveloped and lacks sufficient detail regarding the data sources obtained from the NRSS and Libyan's NCDC. Additional information should be provided on data collection procedures, reporting systems, case definitions, data quality considerations, and any preprocessing steps undertaken before analysis. The authors may find it useful to review the structure and level of methodological detail presented in the following studies as examples of good practice (these references are provided solely as examples of reporting structure and do not need to be cited within the manuscript):

Musah et al. (2020): https://doi.org/10.1016/j.apgeog.2019.102126
Ozius et al. (2021): https://doi.org/10.1007/s13753-021-00369-z

Comments on Figures 3A-3C
From a visualisation and data-storytelling perspective, Figures 3A–3C do not effectively communicate the geographic and temporal burden of diarrhoeal disease. At present, the figures show only the north-western portion of Africa with coloured markers representing affected locations, making interpretation difficult. I strongly recommend substantial revision of these figures:

1. Provide appropriate geographic context. Libya's national boundaries and administrative divisions should be included so that readers can clearly identify the locations being presented.
2. Use a meaningful colour scale. The current colour scheme appears arbitrary and does not effectively communicate disease burden. A sequential colour scale (e.g., white-yellow-red) representing increasing case counts would be more appropriate.
3. If I am reading these numbers correctly – are these absolute case counts? Would it be better to present them in relative terms (incidence rates) instead of in absolute terms?
