# Supplementary material for: Post-flood communicable disease surveillance following the 2023 dam collapse in Eastern Libya
Source: PLOS Glob Public Health. 2026 Aug 20;6(8):e0007124. doi: 10.1371/journal.pgph.0007124 (PMC13492783; doi:10.1371/journal.pgph.0007124)
Supplement: Supplementary file 4 [file pgph.0007124.s005.pdf]

Dear Editor and Reviewers,

Thank you very much for providing us with the opportunity to revise our manuscript and for sending us the constructive comments to further improve our work.

We have carefully reviewed and addressed all the remaining comments from the esteemed Reviewer #4. We believe that this revision has substantially improved the quality, context, and clarity of our manuscript, making it fully ready for publication.

In particular, we have thoroughly integrated the reviewer's suggestions to contextualize the public health impact of the September 2023 Libyan floods within the country's unique environmental, infrastructural, and post-conflict setting. We have also refined our methodology descriptions and overhauled our visual data storytelling.

Reviewer #4

Comment:

Overall Feedback

This manuscript addresses an important public health issue in the aftermath of the September 2023 floods in Libya. However, substantial revisions (major) are required before the manuscript can be considered for publication.

In particular, the introduction and methods sections are currently too generic and would benefit from significant structural revision to better contextualise the study within Libya's unique environmental, infrastructural, and post-conflict setting, while providing greater methodological detail and transparency.

Response:

Thank you for raising these points. We will definitely follow the comments of the esteemed reviewer, considering these comments as an opportunity for the manuscript improvement.

Comment:

Abstract

The abstract could be strengthened by providing additional detail on the study design and quantitative methods employed. Rather than simply stating that the analysis was conducted in R, the authors should briefly describe the ecologic descriptive analytical approach used and the rationale behind it. This would provide readers with a clearer understanding of the study methodology and findings.

Response:

We have updated the methods part of the abstract and added details related to our ecological approach, and the benefit of applying it.

Comment:

Introduction

Would it be appropriate to include Libya's current post-conflict situation as an additional and potentially unique risk factor contributing to the impacts of flood events? Sustained damage resulting from both internal and external conflicts has likely affected critical infrastructure and may have reduced the country's resilience to environmental disasters.

Response:

We have followed this comment and added more information to the introduction to explain the Libyan-specific situation. We believe following this comment has a good impact on the paper.

Comment:

Although the manuscript discusses several broad risk factors for diarrhoeal disease, the discussion remains largely generic. This section would benefit from a stronger focus on Libya-specific determinants of disease transmission. For example, Libya's predominantly arid environment and associated water scarcity may increase reliance on alternative water sources, potentially elevating the risk of diarrhoeal infections. Furthermore, damage to sanitation and wastewater infrastructure during years of conflict may have increased population exposure to waterborne pathogens. Incorporating these contextual factors would help situate the study within the specific environmental and socio-political realities of Libya.

Response:

Thank you for suggesting these important additions. We have incorporated them in the introduction, explaining the impact of the climate as well as the long-lasting conflict on the water supply and resources.

Comment:

The introduction would also benefit from providing greater context regarding the September 10, 2023 flood disaster. While the event was triggered by an extreme weather event, it is important to discuss the broader factors that contributed to the catastrophe. For example, were the affected dams already structurally weakened due to conflict-related damage, inadequate maintenance, ageing infrastructure, or a combination of these factors? Such discussion would provide valuable context that is Libyan centred and help readers better understand why the disaster occurred. Additionally, the names and locations of the two dam structures involved in the disaster should be clearly stated.

Response:

We have added the required information to the introduction section. We also added a few references to the readers to get a deeper understanding of the situation.

Comment:

Methods

The methods section would benefit from the inclusion of a dedicated Study Area subsection describing Libya's geographic, demographic, and socioeconomic characteristics. Such a section would help readers unfamiliar with the country better understand the study context. I also strongly recommend including a map illustrating Libya's administrative boundaries, major population centres, the locations of the affected dams, and the relevant river or watershed systems associated with the flood event. This would substantially improve the geographical context of the study.

Response:

We have added more information on the methods section; however, we failed to get the current administrative boundaries, because these boundaries are quickly changing. We included the available relevant information to the article. For the relevant river and

watershed systems, we have added references of articles that have the required details and were written by specialists. We feel we, as medical professionals, are not well qualified to discuss these details in this paper.

Comment:

More broadly, the methods section is currently underdeveloped and lacks sufficient detail regarding the data sources obtained from the NRSS and Libyan's NCDC. Additional information should be provided on data collection procedures, reporting systems, case definitions, data quality considerations, and any preprocessing steps undertaken before analysis. The authors may find it useful to review the structure and level of methodological detail presented in the following studies as examples of good practice (these references are provided solely as examples of reporting structure and do not need to be cited within the manuscript):

Musah et al. (2020): `https://doi.org/10.1016/j.apgeog.2019.102126`

Ozius et al. (2021): `https://doi.org/10.1007/s13753-021-00369-z`

Response:

We have followed these comments in the previous submission (rev-1). We read these two papers and benefited from them, and used the knowledge from them to improve our work.

Comment:

Comments on Figures 3A–3C

From a visualisation and data-storytelling perspective, Figures 3A–3C do not effectively communicate the geographic and temporal burden of diarrhoeal disease. At present, the figures show only the north-western portion of Africa with coloured markers representing affected locations, making interpretation difficult. I strongly recommend substantial revision of these figures:

Response:

We have modified figure 3 substantially in the previous submission (rev-1), the main changes are:

- Adding another map about respiratory infection.
- Change the values from absolute case counts to incidence rates depending on the local district population.
- We made the size of the circle correspond to the magnitude of the incidence rate

After receiving the reviewer comment, we added a bigger map of the region to clarify the position of the affected area, and made the color also correspond to the magnitude of the incidence rate.

Comment:

[1] Provide appropriate geographic context. Libya's national boundaries and administrative divisions should be included so that readers can clearly identify the locations being presented.

Response:

The current data about the boundaries of Libyan administrative divisions are not fixed. We contacted several authorities in person to find a good source for this information, but we received negative responses. They told us that these data are not currently available because the Libyan administrative divisions are changing dynamically, and there is no clear boundaries assigned by the Ministry of Local Governance. This situation is probably caused by the instability in the country after many political conflicts.

Therefore, we chose not to include old information in our paper since we were not able to provide the current one. However, we updated the figure to show the full map of Libya, and the current one shows the location of the affected area. Moreover, we updated the text to further describe the geographical area and the country boundaries. In addition to that, we cited some specialized articles for more details.

Comment:

[2] Use a meaningful colour scale. The current colour scheme appears arbitrary and does not effectively communicate disease burden. A sequential colour scale (e.g., white–yellow–red) representing increasing case counts would be more appropriate.

Response:

Thank you for this great suggestion, we have followed it.

Comment:

[3] If I am reading these numbers correctly – are these absolute case counts? Would it be better to present them in relative terms (incidence rates) instead of in absolute terms?

Response:

We have followed this comment and calculated the incidence rate, we have also updated the figure description to explicitely mention that these values are incidence rates.
