# Supplementary material for: Post-flood communicable disease surveillance following the 2023 dam collapse in Eastern Libya
Source: PLOS Glob Public Health. 2026 Aug 20;6(8):e0007124. doi: 10.1371/journal.pgph.0007124 (PMC13492783; doi:10.1371/journal.pgph.0007124)
Supplement: Peer review history

# Peer review: Decision Letter 0

**DOI:** 10.1371/journal.pgph.0007124.r001

**Authors:** Helen Howard

8 Mar 2026

PGPH-D-25-03695

Post-Flood Communicable Disease Surveillance Following the 2023 Dam Collapse in Eastern Libya.

PLOS Global Public Health

Dear Dr. Mohsen,

Thank you for submitting your manuscript to PLOS Global Public Health. After careful consideration, we feel that it has merit but does not fully meet PLOS Global Public Health’s publication criteria as it currently stands. Therefore, we invite you to submit a revised version of the manuscript that addresses the points raised during the review process.

The manuscript has been evaluated by three reviewers, and their comments are available below.

The reviewers have raised a number of concerns that need attention. In particular, they request additional information on methodological aspects of the study, improvements to the Introduction and Discussion sections, as well as improvements to the quality of the English language.

Could you please revise the manuscript to carefully address the concerns raised?

Please submit your revised manuscript by Apr 22 2026 11:59PM. If you will need more time than this to complete your revisions, please reply to this message or contact the journal office at globalpubhealth@plos.org. When you're ready to submit your revision, log on to https://www.editorialmanager.com/pgph/ and select the 'Submissions Needing Revision' folder to locate your manuscript file.

Please include the following items when submitting your revised manuscript:

- A letter that responds to each point raised by the editor and reviewer(s). You should upload this letter as a separate file labeled 'Response to Reviewers'.
- A marked-up copy of your manuscript that highlights changes made to the original version. You should upload this as a separate file labeled 'Revised Manuscript with Track Changes'.
- An unmarked version of your revised paper without tracked changes. You should upload this as a separate file labeled 'Manuscript'.

Guidelines for resubmitting your figure files are available below the reviewer comments at the end of this letter.

We look forward to receiving your revised manuscript.

Kind regards,

Helen Howard

Staff Editor

PLOS Global Public Health

Journal Requirements:

1. We ask that a manuscript source file is provided at Revision. Please upload your manuscript file as a .doc, .docx, .rtf or .tex.

2.  Please upload separate figure files in .tif or .eps format. Also, remove the figures from your manuscript file but keep the legends.

For more information about figure files please see our guidelines:

https://journals.plos.org/globalpublichealth/s/figures

https://journals.plos.org/globalpublichealth/s/figures#loc-file-requirements

3. We note that your Data Availability Statement is currently as follows: Data is provided in the manuscript, further inquries will be considered by the authors.

Please confirm at this time whether or not your submission contains all raw data required to replicate the results of your study. Authors must share the “minimal data set” for their submission. PLOS defines the minimal data set to consist of the data required to replicate all study findings reported in the article, as well as related metadata and methods

http://journals.plos.org/globalpublichealth/s/data-availability#loc-minimal-data-set-definition

For example, authors should submit the following data:

- The values behind the means, standard deviations and other measures reported;

- The values used to build graphs;

- The points extracted from images for analysis.

Authors do not need to submit their entire data set if only a portion of the data was used in the reported study.

If your submission does not contain these data, please either upload them as Supporting Information files or deposit them to a stable, public repository and provide us with the relevant URLs, DOIs, or accession numbers. For a list of recommended repositories, please see

http://journals.plos.org/globalpublichealth/s/recommended-repositories

If there are ethical or legal restrictions on sharing a de-identified data set, please explain them in detail (e.g., data contain potentially sensitive information, data are owned by a third-party organization, etc.) and who has imposed them (e.g., an ethics committee). Please also provide contact information for a data access committee, ethics committee, or other institutional body to which data requests may be sent. If data are owned by a third party, please indicate how others may request data access.

4. Some material included in your submission may be copyrighted. According to PLOS’s copyright policy, authors who use figures or other material (e.g., graphics, clipart, maps) from another author or copyright holder must demonstrate or obtain permission to publish this material under the Creative Commons Attribution 4.0 International (CC BY 4.0) License used by PLOS journals. Please closely review the details of PLOS’s copyright requirements here: PLOS Licenses and Copyright. If you need to request permissions from a copyright holder, you may use PLOS's Copyright Content Permission form.

Please respond directly to this email or email the journal office and provide any known details concerning your material's license terms and permissions required for reuse, even if you have not yet obtained copyright permissions or are unsure of your material's copyright compatibility.

Potential Copyright Issues:

a. Figure 3: please (a) provide a direct link to the base layer of the map (i.e., the country or region border shape) and ensure this is also included in the figure legend; and (b) provide a link to the terms of use / license information for the base layer image or shapefile. We cannot publish proprietary or copyrighted maps (e.g. Google Maps, Mapquest) and the terms of use for your map base layer must be compatible with our CC-BY 4.0 license.

Note: if you created the map in a software program like R or ArcGIS, please locate and indicate the source of the basemap shapefile onto which data has been plotted.

If your map was obtained from a copyrighted source please amend the figure so that the base map used is from an openly available source. Alternatively, please provide explicit written permission from the copyright holder granting you the right to publish the material under our CC-BY 4.0 license.

Please note that the following CC BY licenses are compatible with PLOS license: CC BY 4.0, CC BY 2.0 and CC BY 3.0, meanwhile such licenses as CC BY-ND 3.0 and others are not compatible due to additional restrictions.

If you are unsure whether you can use a map or not, please do reach out and we will be able to help you. The following websites are good examples of where you can source open access or public domain maps:

* U.S. Geological Survey (USGS) - All maps are in the public domain. (http://www.usgs.gov)

* PlaniGlobe - All maps are published under a Creative Commons license so please cite “PlaniGlobe, http://www.planiglobe.com, CC BY 2.0” in the image credit after the caption. (http://www.planiglobe.com/?lang=enl)

* Natural Earth - All maps are public domain. (http://www.naturalearthdata.com/about/terms-of-use/)

If the reviewer comments include a recommendation to cite specific previously published works, please review and evaluate these publications to determine whether they are relevant and should be cited. There is no requirement to cite these works unless the editor has indicated otherwise.

Additional Editor Comments (if provided):

[Note: HTML markup is below. Please do not edit.]

Reviewers' comments:

Reviewer's Responses to Questions

**Comments to the Author**

1. Does this manuscript meet PLOS Global Public Health’s publication criteria? Is the manuscript technically sound, and do the data support the conclusions? The manuscript must describe methodologically and ethically rigorous research with conclusions that are appropriately drawn based on the data presented.

Reviewer #1: Partly

Reviewer #2: Yes

Reviewer #3: Partly

**********

2. Has the statistical analysis been performed appropriately and rigorously?

Reviewer #1: Yes

Reviewer #2: Yes

Reviewer #3: No

**********

3. Have the authors made all data underlying the findings in their manuscript fully available (please refer to the Data Availability Statement at the start of the manuscript PDF file)?

The PLOS Data policy requires authors to make all data underlying the findings described in their manuscript fully available without restriction, with rare exception. The data should be provided as part of the manuscript or its supporting information, or deposited to a public repository. For example, in addition to summary statistics, the data points behind means, medians and variance measures should be available. If there are restrictions on publicly sharing data—e.g. participant privacy or use of data from a third party—those must be specified.

Reviewer #1: No

Reviewer #2: No

Reviewer #3: No

**********

4. Is the manuscript presented in an intelligible fashion and written in standard English?

PLOS Global Public Health does not copyedit accepted manuscripts, so the language in submitted articles must be clear, correct, and unambiguous. Any typographical or grammatical errors should be corrected at revision, so please note any specific errors here.

Reviewer #1: Yes

Reviewer #2: Yes

Reviewer #3: Yes

**********

5. Review Comments to the Author

Please use the space provided to explain your answers to the questions above. You may also include additional comments for the author, including concerns about dual publication, research ethics, or publication ethics. (Please upload your review as an attachment if it exceeds 20,000 characters)

Reviewer #1: Abstract

The authors have mentioned only two types of infectious diseases. What about the others?

The conclusion needs to be accurate and should clearly reflect the study findings.

Keywords are missing.

Introduction

The introduction requires a more comprehensive and strengthened literature review relevant to the study.

The impact of floods on infectious diseases and mitigation strategies is missing.

The authors discuss flood-related damages from 2024, while the study focuses on infectious disease events during the 2023 floods.

The need for the study and its novelty should be clearly stated.

Methods

The methods section is insufficient and should be more comprehensive.

The criteria for including reports need to be clearly described.

There is no information on how the validity of the reports was assessed. Are there any references for this?

Which surveillance method was used in this study?

There is no data provided on the fourteen diseases. How were the cases defined, and what were the criteria for selecting these diseases?

How were the incidence rates of the diseases calculated?

Results

The text should clearly refer to the tables.

All fourteen listed diseases should be mentioned in the text, along with their numbers or percentages.

The authors should also report case fatality rates and prevalence per 1,000 population in addition to incidence rates.

Age groups have been divided into less than and more than five years. What about adults, older adults, and pregnant and lactating women (PLWs)?

Why did the authors focus only on the trend of diarrhea? What about trends in other diseases? Which type of analysis was used to assess these trends?

Discussion

The discussion should be structured and developed based on the points raised above.

Reviewer #2: Review of “Post-Flood Communicable Disease Surveillance Following the 2023 Dam Collapse in Eastern Libya. “

To the Authors,

The manuscript “Post-Flood Communicable Disease Surveillance Following the 2023 Dam Collapse in Eastern Libya” provides new information about the health impacts of the 2023 flood and dam collapses in Libya. Because the global climate is warming, more cyclones are expected in the future, and this study provides very useful information for those medical and public health officials who will manage future disasters. The availability of active surveillance data make this study possible, and the authors described the outbreak well in the tables and figures. Several recommendations follow to further strengthen the manuscript.

Major

Context about the collapse of the dams is lacking in the manuscript. A brief description and the findings of Nemnem et al 2025 “How extreme rainfall and failing dams unleashed the Derna flood disaster” (10.1038/s41467-025-59261-9 ) in Nature Communications would give readers important background information and should be added to the manuscript.

More information in the Introduction section about the public health response would be very useful. Were handwashing facilities made available? Latrines? Soap? Methods for point-of-use water disinfection?

Line 48: Written authorization was obtained. Please clarify – was an ethical review part of the process for authorization?

Data generated by the active surveillance system is very important. Equally important would be a clear description of the how the active surveillance system worked. In the Discussion section, the manuscript notes “NRSS depends on official public health officers and doctors to report data from all 94 hospitals and health centers that have the ability to provide emergency health services.” This section should be moved the end of the first paragraph of the Methodology section. Additional information should be added about whether the system is paper-based or electronic and whether “health centers” includes (private) physician offices or whether the system only included public (governmental) health centers. If private physician offices do not submit reports through the NRSS, then the Discussion section should note this as a reason to believe the reported case numbers are an underestimate of actual cases of disease.

Is any information available about the number people hospitalized due to diarrhea, pneumonia, or respiratory illness? Is any information available about the number people seen in emergency departments due to diarrhea, pneumonia, or respiratory illness? If so, that information should be added to the Results section.

The active surveillance system began collecting data only after the flood and dam collapse were not available. The discussion section could note that no “pre-collapase” and “post-collapse” comparisons are possible. However, pre-collapse, there were also cases of diarrhea and respiratory symptoms. Thus, it should be noted in the Discussion section that no “baseline” data are available for comparison.

The Discussion section could be strengthened by adding several sentences about lessons learned to guide public health professionals who will manage future floods elsewhere.

Other comments

Lines 5-8: “Like many Mediterranean and North African countries, Libya is increasingly vulnerable to climate-related extreme events due to aging infrastructure and limited early-warning capacity. Living in flood-prone areas—especially where early warning systems and flood-related education are lacking—greatly increases the risk of flood-related harm [1, 3].”

The text before this provided a general view of flooding and so does the text that follows in line 8-9, so this information specific to Libya is out of place. It would fit better just before line 23: “In Libya, on 10 September 2023, heavy rainfall and strong winds from Tropical Storm Daniel caused the collapse of two dams,”

Lines 8-9: “In 2024, floods caused 5,885 deaths, affected 48 million people, and cost an estimated 32 billion USD [4].” Make it clear that these number are global, not specific to the collapse of the Derna dams.

Line 51: spell out BSC.

Line 58: “diarrhea” instead of “Diarrhea”

Line 59: Instead of “However, the lowest cases were rubella,….” change to “The infections reported least frequently were rubella…”

Line 64: The term disease burden generally refers to the combination of disease frequency and disease severity. Because severity data are not available, the text should be changed from “The biggest burden of the infectious disease after the flood…” to “The most frequent type of infectious disease reported after the flood….”

Line 78=79: Instead of “Diarrhea cases were significantly increased in each day, reaching the peak in day of 42…” change to “The number of diarrhea cases increased steadily until day 42 when case counts peaked and then declined.”

Lines 79-80: “The interpretation of the diarrhea situation was decided as an outbreak which needs an urgent intervention.” Change to “Given the very large number of diarrhea cases, an urgent intervention was needed.”

Lines 81-82 “: .. groundwater in Albayda city, which suffers from the highest number of diarrhea cases in the east.” Change to “groundwater in Albayda city, which experienced the highest number of diarrhea cases in the east.”

Lines 103-104: “This contamination may also have affected crop growing, increasing the risk of further infection spreading.” Change to “This may have had contaminated food crops, increase the risk of food-borne disease transmission.”

Line 109: Were the “underground wells” dug (shallow) wells or deeper drilled well (or “tube wells”)?

Line 118: Change “Cholera” to “cholera” (the disease is not capitalized).

Line 119-120: “This is going well with another survey conducted in China showing no increase in Bacillus Dysentery, Cholera…” The comparison to the pathogens identified in China after a cyclone is not needed and can be deleted. If the authors want to keep this sentence, change the beginning to “This is consistent with a study done in China after a cyclone…”

Line 123: Change “Respiratory infections are probably due to IDP…” to “Crowding due to the large number of internally displaced persons (IDP) probably promoted the transmission of respiratory infections.”

Lines 138-140: Please add information about whether the response included the provision of liquid chlorine (hypochlorite solution) for point-of-use drinking water disinfection.

Line 147-148: Add mental health conditions such as anxiety and depression to the list of problems suffered by IDP.

Reviewer #3: Thank you for bringing to light this evidence and for all of your work writing this piece. I was excited to read the piece, and I think a comparison to a similar season in a prior year could be a really interesting piece.

Here are some of my more detailed comments.

Libya dam collapse article:

Abstract: led not leading

Would love to see a sentence in abstract about implications of this study be more applicable than just to lybia — what does this study say or contribute to in regards to literature about impacts of floods.

Line 1: How do you define most frequent. I think this phrase could be deleted

Line 8: in which geographic region?

Line 16: worsened is not right word to put here

Line 21: Citation needed

Line 25: cities in the northeast of Libya

Line 27: of which nation?

Could present incidence rate per 100,000 instead of million to make more aligned with WHO reports

Fig 1: I have hard time interpreting because I do not know what % of the population is under 5. So do not know what is similar to population structure and what is disproportionately affecting kids <5

I think you should compare the rates of all these diseases to a year before the flood and conduct using a difference in difference analysis in order to make a causal claim. I have no way of knowing in the paper right now if any of these are different than the normal rates in these cities for this time of year.

Fig 3 I do not know why the faceting is the way it is. Can you show the difference in incidence by city please instead of totals?

**********

6. PLOS authors have the option to publish the peer review history of their article (what does this mean?). If published, this will include your full peer review and any attached files.

**Do you want your identity to be public for this peer review?**  If you choose “no”, your identity will remain anonymous but your review may still be made public.

For information about this choice, including consent withdrawal, please see our Privacy Policy.

Reviewer #1: No

Reviewer #2: No

Reviewer #3: **Yes:**  Christopher LeBoa

**********

[NOTE: If reviewer comments were submitted as an attachment file, they will be attached to this email and accessible via the submission site. Please log into your account, locate the manuscript record, and check for the action link "View Attachments". If this link does not appear, there are no attachment files.]

-->Figure Resubmissions:

While revising your submission, we strongly recommend that you use PLOS’s NAAS tool (https://ngplosjournals.pagemajik.ai/artanalysis) to test your figure files. NAAS can convert your figure files to the TIFF file type and meet basic requirements (such as print size, resolution), or provide you with a report on issues that do not meet our requirements and that NAAS cannot fix.

After uploading your figures to PLOS’s NAAS tool - https://ngplosjournals.pagemajik.ai/artanalysis, NAAS will process the files provided and display the results in the "Uploaded Files" section of the page as the processing is complete. If the uploaded figures meet our requirements (or NAAS is able to fix the files to meet our requirements), the figure will be marked as "fixed" above. If NAAS is unable to fix the files, a red "failed" label will appear above. When NAAS has confirmed that the figure files meet our requirements, please download the file via the download option, and include these NAAS processed figure files when submitting your revised manuscript.

# Author comment: Author response to Decision Letter 1

**DOI:** 10.1371/journal.pgph.0007124.r002

17 Apr 2026

Attachment Submitted filename: response-to-reviewers.pdf

# Peer review: Decision Letter 1

**DOI:** 10.1371/journal.pgph.0007124.r003

**Authors:** Xin Sun

10 Jun 2026

PGPH-D-25-03695R1

Post-Flood Communicable Disease Surveillance Following the 2023 Dam Collapse in Eastern Libya.

PLOS Global Public Health

Dear Dr. Mohsen,

Thank you for submitting your manuscript to PLOS Global Public Health. After careful consideration, we feel that it has merit but does not fully meet PLOS Global Public Health’s publication criteria as it currently stands. Therefore, we invite you to submit a revised version of the manuscript that addresses the points raised during the review process.

This manuscript has been evaluated by two experts of the field. They have raised several concerns regarding the methods and the figures. Please address them one by one in your revision.

Please submit your revised manuscript by Aug 09 2026 11:59PM. If you will need more time than this to complete your revisions, please reply to this message or contact the journal office at globalpubhealth@plos.org. When you're ready to submit your revision, log on to https://www.editorialmanager.com/pgph/ and select the 'Submissions Needing Revision' folder to locate your manuscript file.

Please include the following items when submitting your revised manuscript:

- A letter that responds to each point raised by the editor and reviewer(s). You should upload this letter as a separate file labeled 'Response to Reviewers'.
- A marked-up copy of your manuscript that highlights changes made to the original version. You should upload this as a separate file labeled 'Revised Manuscript with Track Changes'.
- An unmarked version of your revised paper without tracked changes. You should upload this as a separate file labeled 'Manuscript'.

Guidelines for resubmitting your figure files are available below the reviewer comments at the end of this letter.

As the corresponding author, your ORCID iD is verified in the submission system and will appear in the published article. PLOS supports the use of ORCID, and we encourage all coauthors to register for an ORCID iD and use it as well. Please encourage your coauthors to verify their ORCID iD within the submission system before final acceptance, as unverified ORCID iDs will not appear in the published article. *Only*  the individual author can complete the verification step; PLOS staff *cannot*  verify ORCID iDs on behalf of authors.

We look forward to receiving your revised manuscript.

Kind regards,

Xin Sun, PhD

Staff Editor

PLOS Global Public Health

**Journal Requirements:**

If the reviewer comments include a recommendation to cite specific previously published works, please review and evaluate these publications to determine whether they are relevant and should be cited. There is no requirement to cite these works unless the editor has indicated otherwise.

**Additional Editor Comments (if provided):**

[Note: HTML markup is below. Please do not edit.]

Reviewers' comments:

Reviewer's Responses to Questions

**Comments to the Author**

1. If the authors have adequately addressed your comments raised in a previous round of review and you feel that this manuscript is now acceptable for publication, you may indicate that here to bypass the “Comments to the Author” section, enter your conflict of interest statement in the “Confidential to Editor” section, and submit your "Accept" recommendation.

Reviewer #2: All comments have been addressed

Reviewer #4: (No Response)

**********

2. Does this manuscript meet PLOS Global Public Health’s publication criteria? Is the manuscript technically sound, and do the data support the conclusions? The manuscript must describe methodologically and ethically rigorous research with conclusions that are appropriately drawn based on the data presented.

Reviewer #2: Yes

Reviewer #4: Partly

**********

3. Has the statistical analysis been performed appropriately and rigorously?

Reviewer #2: N/A

Reviewer #4: Yes

**********

4. Have the authors made all data underlying the findings in their manuscript fully available (please refer to the Data Availability Statement at the start of the manuscript PDF file)?

The PLOS Data policy requires authors to make all data underlying the findings described in their manuscript fully available without restriction, with rare exception. The data should be provided as part of the manuscript or its supporting information, or deposited to a public repository. For example, in addition to summary statistics, the data points behind means, medians and variance measures should be available. If there are restrictions on publicly sharing data—e.g. participant privacy or use of data from a third party—those must be specified.

Reviewer #2: Yes

Reviewer #4: Yes

**********

5. Is the manuscript presented in an intelligible fashion and written in standard English?

PLOS Global Public Health does not copyedit accepted manuscripts, so the language in submitted articles must be clear, correct, and unambiguous. Any typographical or grammatical errors should be corrected at revision, so please note any specific errors here.

Reviewer #2: Yes

Reviewer #4: Yes

**********

6. Review Comments to the Author

Please use the space provided to explain your answers to the questions above. You may also include additional comments for the author, including concerns about dual publication, research ethics, or publication ethics. (Please upload your review as an attachment if it exceeds 20,000 characters)

**Reviewer #2:**  The authors have incorporated suggested revisions, and the overall quality of the manuscript is now acceptable.

**Reviewer #4:**  Overall Feedback

This manuscript addresses an important public health issue in the aftermath of the September 2023 floods in Libya. However, substantial revisions (major) are required before the manuscript can be considered for publication. In particular, the introduction and methods sections are currently too generic and would benefit from significant structural revision to better contextualise the study within Libya's unique environmental, infrastructural, and post-conflict setting, while providing greater methodological detail and transparency.

Abstract

The abstract could be strengthened by providing additional detail on the study design and quantitative methods employed. Rather than simply stating that the analysis was conducted in R, the authors should briefly describe the ecologic descriptive analytical approach used and the rationale behind it. This would provide readers with a clearer understanding of the study methodology and findings.

Introduction

Would it be appropriate to include Libya's current post-conflict situation as an additional and potentially unique risk factor contributing to the impacts of flood events? Sustained damage resulting from both internal and external conflicts has likely affected critical infrastructure and may have reduced the country's resilience to environmental disasters.

Although the manuscript discusses several broad risk factors for diarrhoeal disease, the discussion remains largely generic. This section would benefit from a stronger focus on Libya-specific determinants of disease transmission. For example, Libya's predominantly arid environment and associated water scarcity may increase reliance on alternative water sources, potentially elevating the risk of diarrhoeal infections. Furthermore, damage to sanitation and wastewater infrastructure during years of conflict may have increased population exposure to waterborne pathogens. Incorporating these contextual factors would help situate the study within the specific environmental and socio-political realities of Libya.

The introduction would also benefit from providing greater context regarding the September 10, 2023 flood disaster. While the event was triggered by an extreme weather event, it is important to discuss the broader factors that contributed to the catastrophe. For example, were the affected dams already structurally weakened due to conflict-related damage, inadequate maintenance, ageing infrastructure, or a combination of these factors? Such discussion would provide valuable context that is Libyan centred and help readers better understand why the disaster occurred. Additionally, the names and locations of the two dam structures involved in the disaster should be clearly stated.

Methods

The methods section would benefit from the inclusion of a dedicated Study Area subsection describing Libya's geographic, demographic, and socioeconomic characteristics. Such a section would help readers unfamiliar with the country better understand the study context. I also strongly recommend including a map illustrating Libya's administrative boundaries, major population centres, the locations of the affected dams, and the relevant river or watershed systems associated with the flood event. This would substantially improve the geographical context of the study.

More broadly, the methods section is currently underdeveloped and lacks sufficient detail regarding the data sources obtained from the NRSS and Libyan’s NCDC. Additional information should be provided on data collection procedures, reporting systems, case definitions, data quality considerations, and any preprocessing steps undertaken before analysis. The authors may find it useful to review the structure and level of methodological detail presented in the following studies as examples of good practice (these references are provided solely as examples of reporting structure and do not need to be cited within the manuscript):

Musah et al. (2020): https://doi.org/10.1016/j.apgeog.2019.102126

Ozius et al. (2021): https://doi.org/10.1007/s13753-021-00369-z

Comments on Figures 3A–3C

From a visualisation and data-storytelling perspective, Figures 3A–3C do not effectively communicate the geographic and temporal burden of diarrhoeal disease. At present, the figures show only the north-western portion of Africa with coloured markers representing affected locations, making interpretation difficult. I strongly recommend substantial revision of these figures:

[1] Provide appropriate geographic context. Libya's national boundaries and administrative divisions should be included so that readers can clearly identify the locations being presented.

[2] Use a meaningful colour scale. The current colour scheme appears arbitrary and does not effectively communicate disease burden. A sequential colour scale (e.g., white–yellow–red) representing increasing case counts would be more appropriate.

[3] If I am reading these numbers correctly – are these absolute case counts? Would it be better to present them in relative terms (incidence rates) instead of in absolute terms?

**********

7. PLOS authors have the option to publish the peer review history of their article (what does this mean?). If published, this will include your full peer review and any attached files.

**Do you want your identity to be public for this peer review?**  If you choose “no”, your identity will remain anonymous but your review may still be made public.

For information about this choice, including consent withdrawal, please see our Privacy Policy.

Reviewer #2: No

Reviewer #4: No

**********

[NOTE: If reviewer comments were submitted as an attachment file, they will be attached to this email and accessible via the submission site. Please log into your account, locate the manuscript record, and check for the action link "View Attachments". If this link does not appear, there are no attachment files.]

-->Figure Resubmissions:

While revising your submission, we strongly recommend that you use PLOS’s NAAS tool (https://ngplosjournals.pagemajik.ai/artanalysis) to test your figure files. NAAS can convert your figure files to the TIFF file type and meet basic requirements (such as print size, resolution), or provide you with a report on issues that do not meet our requirements and that NAAS cannot fix.

After uploading your figures to PLOS’s NAAS tool - https://ngplosjournals.pagemajik.ai/artanalysis, NAAS will process the files provided and display the results in the "Uploaded Files" section of the page as the processing is complete. If the uploaded figures meet our requirements (or NAAS is able to fix the files to meet our requirements), the figure will be marked as "fixed" above. If NAAS is unable to fix the files, a red "failed" label will appear above. When NAAS has confirmed that the figure files meet our requirements, please download the file via the download option, and include these NAAS processed figure files when submitting your revised manuscript.

Attachment Submitted filename: PGPH-D-25-03695R1 - Reviewer Comments.pdf

# Author comment: Author response to Decision Letter 2

**DOI:** 10.1371/journal.pgph.0007124.r004

10 Jul 2026

Attachment Submitted filename: response-to-reviewer.pdf

# Peer review: Decision Letter 2

**DOI:** 10.1371/journal.pgph.0007124.r005

**Authors:** Julia Robinson

30 Jul 2026

Post-Flood Communicable Disease Surveillance Following the 2023 Dam Collapse in Eastern Libya.

PGPH-D-25-03695R2

Dear Dr. Mohsen,

We are pleased to inform you that your manuscript 'Post-Flood Communicable Disease Surveillance Following the 2023 Dam Collapse in Eastern Libya.' has been provisionally accepted for publication in PLOS Global Public Health.

Before your manuscript can be formally accepted you will need to complete some formatting changes, which you will receive in a follow up email. A member of our team will be in touch with a set of requests.

Please note that your manuscript will not be scheduled for publication until you have made the required changes, so a swift response is appreciated.

IMPORTANT: The editorial review process is now complete. PLOS will only permit corrections to spelling, formatting or significant scientific errors from this point onwards. Requests for major changes, or any which affect the scientific understanding of your work, will cause delays to the publication date of your manuscript.

If your institution or institutions have a press office, please notify them about your upcoming paper to help maximize its impact. If they'll be preparing press materials, please inform our press team as soon as possible -- no later than 48 hours after receiving the formal acceptance. Your manuscript will remain under strict press embargo until 2 pm Eastern Time on the date of publication. For more information, please contact globalpubhealth@plos.org.

Thank you again for supporting Open Access publishing; we are looking forward to publishing your work in PLOS Global Public Health.

Best regards,

Julia Robinson

Executive Editor

PLOS Global Public Health

***********************************************************

Reviewer Comments (if any, and for reference):

Reviewer's Responses to Questions

**Comments to the Author**

1. If the authors have adequately addressed your comments raised in a previous round of review and you feel that this manuscript is now acceptable for publication, you may indicate that here to bypass the “Comments to the Author” section, enter your conflict of interest statement in the “Confidential to Editor” section, and submit your "Accept" recommendation.

Reviewer #2: All comments have been addressed

**********

2. Does this manuscript meet PLOS Global Public Health’s publication criteria? Is the manuscript technically sound, and do the data support the conclusions? The manuscript must describe methodologically and ethically rigorous research with conclusions that are appropriately drawn based on the data presented.

Reviewer #2: Yes

**********

3. Has the statistical analysis been performed appropriately and rigorously?

Reviewer #2: Yes

**********

4. Have the authors made all data underlying the findings in their manuscript fully available (please refer to the Data Availability Statement at the start of the manuscript PDF file)?

The PLOS Data policy requires authors to make all data underlying the findings described in their manuscript fully available without restriction, with rare exception. The data should be provided as part of the manuscript or its supporting information, or deposited to a public repository. For example, in addition to summary statistics, the data points behind means, medians and variance measures should be available. If there are restrictions on publicly sharing data—e.g. participant privacy or use of data from a third party—those must be specified.

Reviewer #2: Yes

**********

5. Is the manuscript presented in an intelligible fashion and written in standard English?

PLOS Global Public Health does not copyedit accepted manuscripts, so the language in submitted articles must be clear, correct, and unambiguous. Any typographical or grammatical errors should be corrected at revision, so please note any specific errors here.

Reviewer #2: Yes

**********

6. Review Comments to the Author

Please use the space provided to explain your answers to the questions above. You may also include additional comments for the author, including concerns about dual publication, research ethics, or publication ethics. (Please upload your review as an attachment if it exceeds 20,000 characters)

Reviewer #2: Well done.

**********

7. PLOS authors have the option to publish the peer review history of their article (what does this mean?). If published, this will include your full peer review and any attached files.

**Do you want your identity to be public for this peer review?**  If you choose “no”, your identity will remain anonymous but your review may still be made public.

For information about this choice, including consent withdrawal, please see our Privacy Policy.

Reviewer #2: No

**********
